# LC-MS analysis and antioxidant, antibacterial, and antidiabetic activity of *Jumli Marshi* rice from Nepal: An *in vitro and in silico* investigation to validate their potential as a functional food

**DOI:** 10.1371/journal.pone.0319338

**Published:** 2025-03-10

**Authors:** Ram Kishor Yadav, Rekha Bhandari, Harish Babu P C, Prabhat Kumar Jha, Bipindra Pandey, Sindhu KC, Siddha Raj Upadhaya, Sushil Panta, Sajan Lal Shyaula, Khem Raj Joshi

**Affiliations:** 1 School of Health and Allied Sciences, Pokhara University, Pokhara, Nepal; 2 Honeychem Pharma Analytical Services Private Limited, Bangalore, Karnataka, India; 3 Department of Pharmacy, Madan Bhandari Academy of Health Science, Hetauda, Nepal; 4 Central Department of Chemistry, Tribhuvan University, Kathmandu, Nepal; 5 Nepal Academy of Science and Technology (NAST), Lalitpur, Nepal; Kwara State University, NIGERIA

## Abstract

*Jumli Marshi (J. Marshi)*, a native rice cultivar in Nepal, is gaining popularity owing to its health benefits for obesity, hypertension, and diabetes. However, scientific evidence verifying its therapeutic potential is lacking until November 2024. Therefore, we aimed to characterize the phytoconstituents and evaluate the antioxidant, antibacterial, and antidiabetic properties of *J. Marshi,* along with its ADME toxicity profile, using both *in vitro* and *in silico* approaches. Liquid chromatography-mass spectrometry analysis of a 70% methanol extract from *J. Marshi* identified ten plant-based compounds, including phenolic acids, flavonoids, and γ-oryzanol. The extract exhibited significant antioxidant properties, neutralizing DPPH free radicals with a fifty-percentage inhibitory concentration (IC_50_) of 42.65 ± 3.9 µg/mL, compared to ascorbic acid’s IC_50_ of 4.12 ± 0.7 µg/mL. It also showed antibacterial activity against *Staphylococcus aureus*, with a zone of inhibition (ZOI) ranging from 7 to 11 mm and a minimum inhibitory concentration (MIC) of 1.56 mg/mL, compared to standard antibiotics meropenem (ZOI: 24 ± 1.6 mm; MIC: 1.56 mg/mL). The enzymatic assay demonstrated that the *J. Marshi* extract inhibits fifty percent of enzyme activity at a concentration (EC_50_) of > 1000 µg/mL for *α*-amylase and 250 ± 2.5 µg/mL for *α*-glucosidase, in contrast to the standard acarbose, exhibiting an EC_50_ of 35.5 ± 1.5 µg/mL for *α*-amylase and 189.5 ± 1.9 µg/mL for *α*-glucosidase. *In silico* docking studies revealed strong interactions of rice phytoconstituents with target protein catalytic residues, particularly gamma-oryzanol for *α*-amylase (−10.0 kcal/mol) and chlorogenic acid for *α*-glucosidase (−7.7 kcal/mol), compared to acarbose (−6.9 to −7.1 kcal/mol). ADME toxicity analysis suggested that tricin and gamma-oryzanol had the best drug-likeness and safety profiles. To our knowledge, this is the first study to reveal the presence of bioactive phenolic acids and flavonoids. Furthermore, it offers scientific evidence supporting significant antioxidant and *α*-glucosidase-inhibitory properties, confirming the potential applications of *J. Marshi* rice as a functional food used for the management of diabetes.

## 1. Introduction

Foods enriched with biologically active ingredients are recognized as functional foods owing to their physiological and biochemical associations that benefit human health [1]. For example, curcumin of turmeric, ascorbic acid of citric fruits, phenolics of ginger, anthocyanin of blackberry, grapes, and strawberry; lycopene in tomato, watermelon, and guava; and carotenoids of carrot are the few bioactive compounds containing functional foods [2]. Over the past few decades, the use of functional foods as complementary therapies for disease prevention and management has rapidly increased [1]. It is frequently practiced to promote health in cases where patients seek relief from symptoms associated with chronic illnesses, including diabetes and hypertension [3]. For instance, the American Diabetic Association recommends a Mediterranean diet rich in polyphenols for individuals to prevent and manage Type-2 diabetes mellitus [4].

Functional foods containing bioactive compounds beyond basic nutrients, such as polyphenols, flavonoids, vitamins, carotenoids, and terpenoids, serve as antioxidants that play a vital role in combating oxidative stress [3]. These compounds reduce the generation of reactive oxygen species (ROS) and free radicals, including hydroxyl (OH), peroxyl, superoxide (O_2_^-^), and hydrogen peroxide (H_2_O_2_) [5]. Consequently, they offer protection against the development of various serious health conditions, including diabetes, aging, cancer, atherosclerosis, ulcers, gastrointestinal disease, neurodegenerative disorders, coronary heart disease, hypertension, and hepatotoxicity [6,7].

Rice is one of the major grain crops consumed by more than half of the global population [8], and advancing its nutritional and therapeutic aspects could significantly impact global health. In particular, pigmented rice, such as red, black, brown, and purple rice, are rich in beneficial compounds, including p-coumaric acid, isorhamnetin, tricin, gallic acid, ferulic acid, cinnamic acid, protocatechuic acid, tocopherols, γ-oryzanols, anthocyanin, apigenin, and luteolin [8–10]. The incorporation of these pigmented rice varieties into daily diets meets the criteria for functional foods [2,3] because of their therapeutic potential, which encompasses antioxidant, antidiabetic, anti-inflammatory, anti-obesity, and anti-hypertensive properties [9,11].

Nepal’s diverse altitude, topography, and climate facilitate the growth of a wide range of rice (*Oryza sativa*) varieties [12], including fascinating and precious pigmented *J. Marshi* rice (*Oryza sativa* var. japonica), which cost approximately NRs. 350 per kilogram. *J. Marshi* is cultivated only in the Jumla district of Nepal, especially in Chumchaur, the world’s highest growing elevation of approximately 3050 m above sea level [12,13]. *J. Marshi* rice contains a cold-tolerant gene that enables it to grow at a chilling temperature of approximately 4 °C [12,13]. More than six months are required to complete the one-growing season. When transplanted in the month of Jestha (June), it is ready for harvest by Kartik (November) [13]. *J. Marshi* is recognized by its red color, with a black or white husk cover. Analysis of nutritional content showed that *J. Marshi* is a valuable source of nutrients. It contains 2% fiber, 9.68% proteins, and significant amounts of minerals such as iron (0.57 mg/100 g), calcium (66.70 mg/100 g), and phosphorous (57.54 mg/100 g). Additionally, it has a high carbohydrate content (72.74%) [13]. This rice variety is commonly consumed as part of a meal with lentils, vegetables, and pickles, known as “Dal Vat.” It is also a key ingredient in the preparation of the traditional Nepalese bread called “Sel Roti.” When eaten with milk and ghee, *J. Marshi* are considered flavorful, appetizing, and nutritionally beneficial [13].

In global food practice scenarios, the consumption of white rice has been linked to a higher risk of developing diabetes; hence, patients with diabetes often limit or avoid their intake owing to potential exacerbation of diabetic complications [14–16]. Interestingly, *J. Marshi* is consumed by diabetic and obese patients in the Gandaki and Bagmati provinces of Nepal, with an immense belief in its beneficial effects in diabetes and obesity mitigation [13]. Previous studies of pigmented rice cultivars have reported that anthocyanins, flavonoids, and phenolic compounds have significant antidiabetic properties [9,11]. More precisely, Shimoda et al. [16] demonstrated significant antidiabetic activity of purple rice extract by assessing its inhibitory potential on *α*-amylase and *α*-glucosidase, yielding IC_50_ values of 135 and 409 μg/mL, respectively. Similarly, another research on red, brown, and purple rice bran reported superior *α*-glucosidase inhibitory activity of purple rice bran and red rice bran, with an IC_50_ of 8.44 μg/mL and 41.4 μg/mL, respectively, compared to the standard acarbose, which had an IC_50_ of greater than 500 μg/mL [11].

**Fig 1:**
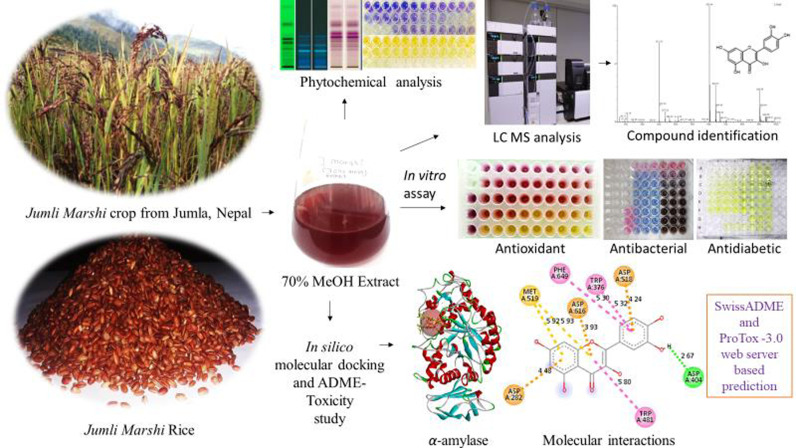
An overview of methods and protocols implemented for the investigation of native rice cultivar, *J.* *Marshi* from Nepal.

These findings sparked our curiosity in investigating the native red-colored *J. Marshi* rice cultivar. Due to the lack of scientific evidence regarding its bioactive phytoconstituents and bioactivities until November 2024, our research aimed to clarify consumer claims about its benefits for diabetes mitigation. The present study seeks to establish *J. Marshi* as a functional food by assessing its phytoconstituents and investigating its antioxidant, antibacterial, and antidiabetic properties for the first time. Additionally, we plan to conduct *in silico* molecular docking studies and assess ADME toxicity parameters to support our *in vitro* findings and elucidate the mechanism of action responsible for its biological activity, as presented in Fig 1.

## 2. Materials and methods

### 2.1. Chemicals and bacterial strain

This study used various chemicals and materials from various suppliers. These included ascorbic acid and acetonitrile from Merck, India; formic acid, aluminum chloride (AlCl_3_), and sodium carbonate from SRL, India; d-glucose, sulfuric acid (H_2_SO_4_), methanol (MeOH), and phenol from Thermo Fisher Scientific, India; gallic acid from Loba Chemie, India; Mueller Hinton agar, meropenem disc, and quercetin dihydrate from HiMedia, India; Folin-Ciocalteu phenol reagent from SD Fine-Chem Ltd, India; and 2,2-diphenyl-1-picrylhydrazyl (DPPH), 2-chloro-4-nitrophenyl-α-D-maltotrioside (CNPG3), *p*-nitrophenyl-α-D-glucopyranoside (*p*NPG), acarbose, α-amylase, and α-glucosidase (Sigma-Aldrich, USA). Bacterial strains were obtained from Manipal Teaching Hospital in Pokhara, Nepal. These strains included *Staphylococcus aureus* (ATCC 11238), *Klebsiella pneumoniae* (ATCC 70065), *Pseudomonas aeruginosa* (ATCC 9027), and *Escherichia coli* (ATCC 11386).

### 2.2. Extraction

The *J. Marshi* rice was collected from a farmer in the Jumla District, Nepal. Rice was extracted using a maceration protocol as previously described [17]. MeOH (70%) was used for the maceration of raw *J. Marshi* rice at a rice-to-solvent ratio of 1:10 for 4 h at 55 °C in a digital water bath (JA-LE-8210; Jaincolab, India), followed by 68 h at room temperature. The contents were filtered through a thick cotton bed and evaporated using a rotary evaporator (Biobase RE-2000B, China) to obtain the crude slurry extract. Subsequently, a vacuum desiccator was used to obtain the dry *J. Marshi* rice extract. The extraction yield was calculated as follows:


$$\text{Extraction yield }\left(\text{\%}\right)\text{= }\left(\text{weight of dry extract/weight of rice used for extraction}\right)\text{×100}.$$

### 2.3. Phytochemical analysis

#### 2.3.1. Qualitative.

The presence of phytochemicals, such as alkaloids, phenols, flavonoids, tannins, anthocyanins, carbohydrates, coumarins, saponins, terpenoids, quinines, steroids, and proteins in *J. Marshi* extracts were tested as previous methods [18,19].

#### 2.3.2. Quantitative.

**2.3.2.1. Total phenolic content (TPC):** Total phenolic content (TPC) of *J. Marshi* rice extract was determined by spectrophotometry using the Folin-Ciocalteu reagent method [17]. The process involved mixing 100 μL of extract (1000 µg/mL in methanol), 6 mL distilled water, and 0.5 mL of 2 N Folin-Ciocalteu phenol reagent with a vortex mixer (BJPX-VW, Biobase) for 10 s. Subsequently, after 5 minutes, 1.5 mL of 7.5% sodium carbonate and 1.9 mL of distilled water (1.5 mL) were added, mixed, and incubated for 2 h in the dark. Gallic acid (31.25 μg/mL–500 μg/mL) served as the reference phenolic compound for the calibration curve and underwent the same treatment as the extract. The absorbance of the solution was measured at 750 nm using an Agilent Cary 60 single-beam UV-VIS spectrophotometer (Malaysia). A blank solution was prepared by substituting the extract with 100 μL methanol and treating it identically. The average absorbance of triplicate samples was used, and TPC was reported as milligrams of gallic acid equivalents (mg of GAE) per gram of extract.

**2.3.2.2. Total flavonoid content (TFC):** Total flavonoid content (TFC) of *J. Marshi* rice extracts were analyzed using the aluminum chloride (AlCl_3_) method [17]. Equal volumes (2 mL) of the AlCl_3_ solution (2% in methanol) and rice extract (100 µg/mL in methanol) were combined and incubated at room temperature for 10 min. Quercetin, which served as a reference flavonoid, was used to create a calibration curve at concentrations ranging from 12.5 to 100 µg/mL following the same procedure as the extract. The absorbance of the solution was measured against a blank (methanol extract) at 415 nm using a single-beam UV-VIS spectrophotometer. TFC was calculated based on the average absorbance of three replicates per sample and expressed as milligrams of quercetin equivalent (mg of QE) per gram of extract.

**2.3.2.3. Total carbohydrate content (TCC):** Total carbohydrate content (TCC) in *J. Marshi* rice extract was determined using a spectrophotometric method following the procedure outlined in our previous study [17]. The process involved combining 1 mL of rice extract (250 µg/mL concentration) with (0.5 mL phenol solution and 2.5 mL H_2_SO_4_. The mixture was incubated at room temperature for 30 min, after which absorbance was measured at 490 nm. A blank containing distilled water instead of extract was used for comparison. D-glucose served as the standard carbohydrate, with concentrations ranging from 12.5 to 200 µg/mL, and was used to create a calibration curve. TCC was expressed in milligrams of d-glucose equivalent (GE) per gram of rice extract.

### 2.4. TLC profiling

Thin-layer chromatography (TLC) was conducted as described in our previous study [17]. A microcapillary tube (Remediolife, India) was used to apply a 1 mg/mL solution of particle-free rice extract to the silica gel 60 F_254_ plates. The plates, which were loaded with the extract band, were placed in a glass beaker that had been saturated with a solvent mixture consisting of chloroform, methanol, and water in a 7:3:0.5 ratio. After the development, the plates were dried in hot air. Subsequently, they were examined under UV light at wavelengths of 254 and 365 nm, followed by immersion in DPPH solution (500 *µ*M) for further analysis.

### 2.5. LC MS analysis

An LC-ESI/MS system was used to examine the 70% methanol extract of *J. Marshi* [20]. Liquid chromatography was conducted using a Shimadzu HPLC (LC-MS 2020) coupled with a Waters XBridge C_18_ column (50 × 4.6 mm, 3.5 µ) at 35 °C. The mobile phases consisted of 0.1% formic acid in water (A) and pure acetonitrile (B), flowing at 1.2 mL/min in a linear gradient as follows: (Time- %A/% B): 0 min−85/15, 6 min−25/75, and 11–15 min−85/15. The analysis used 5 µL of particle-free extract at a concentration of 1 mg/mL. An integrated single quadrupole mass analyzer (LC-MS 2020) was used to perform mass spectrometric detection using electron spray ionization in both positive (ESI^+^) and negative (ESI^−^) modes, scanning from m/z 0–1000. The MS source parameters were set as follows: ESI capillary voltage, 3.03 KV; cone voltage, 13 V; source temperature, 118 °C; desolvation temperature, 246 °C; and gas flow rate: desolation, 500 L/h; and cone, 50 L/h.

### 2.6. *In vitro* biological activity

#### 2.6.1. Antioxidant activity.

The antioxidant properties of the rice extracts were evaluated using the DPPH^•^ scavenging method [18]. Specifically, 1.5 mL of various concentrations were combined with an equal volume of freshly prepared DPPH^•^ methanolic solution (100 µM) on a titer plate (P-3.5-RD-48, China). The mixture was then incubated in the dark at ambient temperature for 30 min, after which absorbance was measured at 517 nm. This measurement was compared against a DPPH control (2 mL methanol substituted for the extract) and methanol blank. Ascorbic acid (0.6125–10 µg/mL) was used as a standard antioxidant for the positive control. The experiment was conducted in triplicate and the average absorbance was used to determine the percentage of DPPH^•^ scavenging activity. Strong antioxidant activity is indicated by a reduction in absorbance, characterized by a color change from purple to pale yellow.

% DPPH^•^ radical scavenging = [(A_0_-A_1_)/A_0_] × 100, where A_0_ represents the absorbance of the DPPH control and A_1_ represents the absorbance of the sample or positive control.

The antioxidant capacity of each extract sample and ascorbic acid is represented by the IC_50_ value (average ± standard deviation). To determine IC_50_, a linear graph depicting the percentage of DPPH scavenging against the concentration of the extract and ascorbic acid was employed.

#### 2.6.2. Antibacterial activity.

The antimicrobial properties of *J. Marshi* extracts were assessed against four bacterial strains obtained from the American Type Culture Collection (ATCC). These strains included *Staphylococcus aureus* (ATCC 11238), *Klebsiella pneumoniae* (ATCC 70065), *Pseudomonas aeruginosa* (ATCC 9027), and *Escherichia coli* (ATCC 11386). The evaluation employed well diffusion and broth microdilution techniques, following the methodology described in a previous study [17].

**2.6.2.1. Well diffusion assay:** Petri dishes and Muller-Hinton agar (MHA) were sterilized for 15 min in an autoclave (HV-110-AC, HOVERLABS) maintained at temperature (121 ºC) and pressure (15 PSI) and then petri plates were filled with MHA using aseptic techniques. Once the culture media solidified, standard bacterial suspensions (0.5 McFarland) were spread across the entire MHA plate surface. Five wells were created in each plate using a sterile 6 mm tip. To seal the well bases, 20 μL melted MHA was added. Various *J. Marshi* extract (100 μL) at concentrations of 25, 50, and 100 mg/mL in sterile water was added to the respective wells. The plates were then incubated for 48 h at 37 °C. Sterile water served as a negative control, while a meropenem 10 μg disc was used as a positive control. Following the 48-hour incubation period, the clear zone of inhibition (ZOI) surrounding the wells was measured in millimeters (mm).

**2.6.2.2. Broth microdilution assay:** The sensitive bacterial strain against the *J. Marshi* extract was further analyzed for its potency in terms of minimum inhibitory concentration (MIC) using the broth microdilution method [21] with slight modifications. Briefly, 100 µL of different extract concentrations (7.8125–1000 µg/mL) and meropenem (0.78125–100 µg/mL) were treated with 100 µL of *S. aureus* suspension (0.5 McFarland standard) on a 96-well titer plate (781960, Brandtech Scientific) and then incubated for 24 h at 37 °C. Additionally, resazurin dye was added to each well to monitor *S. aureus* growth (color change from blue to pink). The minimum concentration of treatment showing no growth on visual observation (blue color) was considered as the MIC against the negative control containing Muller-Hinton broth instead of extract or antibiotics.

#### 2.6.3. Antidiabetic activity.

**2.6.3.1. *α*-Amylase inhibition assay:** The *α*-amylase inhibitory activity was assessed using a previously described protocol [22]. In a 96-well plate (781960, Brandtech Scientific), 20 µL *J. Marshi* extract at various concentrations (10–1000 µg/mL) was combined with 80 µL of porcine pancreatic *α*-amylase enzyme (1.5 U/mL in 50 mM phosphate-buffer saline, pH 7.0). The mixture was incubated for 15 min at 37 °C. Subsequently, 375 µM 2-chloro-4-nitrophenyl-α-D-maltotrioside (CNPG3) was introduced as a substrate to initiate the enzymatic reaction, which proceeded for an additional 15 min at 37 °C. The absorbance of the resulting product was measured at 405 nm using a microplate reader (ELx808 Bio Tek). Inhibitory activity was determined using the following equation:


$$\alpha \text{-Amylase inhibition }\left(\text{\%}\right)\text{= }\left({\text{A}}_{\text{control}}{\text{- A}}_{\text{sample}}{\text{/A}}_{\text{control}}\right)\text{×100}$$

where A represents the absorbance of the sample and the control.

**2.6.3.2. *α*-Glucosidase inhibition assay:** The *α*-glucosidase inhibitory activity was assessed using a previously established protocol [23,24]. In a 96-well plate, 20 µL of *J. Marshi* extract, ranging in concentration from 10 to 1000 µg/mL, was combined with 80 µL porcine pancreatic α-glucosidase enzyme (1.5 U/mL in 50 mM phosphate-buffer saline, pH 7.0). The mixture was incubated for 15 min at 37 °C. Subsequently, *p*-nitrophenyl-α-D-glucopyranoside (*p*NPG) (375 µM) was introduced as a substrate to initiate the enzymatic reaction, which proceeded for 15 min at 37 °C. The absorbance of the resulting product was measured at 405 nm using a microplate reader (ELx808 Bio Tek). Inhibitory activity was determined using the following calculation:


$$\alpha \text{-Glucosidase inhibition }\left(\text{\%}\right)\text{= }\left({\text{A}}_{\text{control}}{\text{- A}}_{\text{sample}}{\text{/A}}_{\text{control}}\right)\text{×100}$$

where A represents the absorbance of the sample and control.

### 2.7. *In silico* studies

#### 2.7.1. Molecular docking.

**2.7.1.1. Design of ligands and proteins:** In this molecular docking investigation, *α*-amylase and *α*-glucosidase were chosen as target host proteins for the analysis of antidiabetic potential, as described previously [25–27]. The three-dimensional crystal structures of these proteins (PDB IDs: 4W93 and 5KZW) [28–30] were obtained from the RSCB Protein Data Bank server (https://www.rcsb.org/). Similarly, the phytochemicals were identified through LC-MS analysis of *J. Marshi* extracts were used as guest ligands. The three-dimensional structures of these phytoconstituents, along with the acarbose standard, were obtained from the PubChem database in SDF format and subsequently converted to PDB format using the BIOVIA Discovery Studio Visualizer. The proteins and ligands were then purified and optimized by removing the extraneous components, incorporating essential polar hydrogen atoms, and adding Kollman charge. Finally, they were transformed into pdbqt files using the AutoDock 1.5.6. software.

**2.7.1.2. Validation of target protein:** The accuracy and quality of the selected target proteins were determined by Ramachandran plot using the computational PROCHECK tool (https://saves.mbi.ucla.edu/) as per our previous method [31].

**2.7.1.3. Active site determination and docking process:** Virtual docking was performed using AutoDock Vina, version 1.5.7. For *α*-amylase, a 3D grid box measuring 20 × 20 × 20 was employed with the coordinates x = −9.6, y = 4.4, and z = −22.9, and a spacing of 0.375 Å. Similarly, for *α*-glucosidase, a 20 × 20 × 20 grid box was used with the coordinates x = −13.7, y = −19.6392, and z = −31.94, and the same spacing of 0.375 Å. These grid boxes encompassed all active site amino acid residues within the enzyme catalytic pocket. Following the docking process, BIOVIA Discovery Studio Visualizer 2020 was used to examine the interactions between the docked proteins and ligands [20].

**2.7.1.4. Docking protocol validation:** The accuracy of the docking process was validated by estimating the root mean square deviation (RMSD) using PyMol 2.5.2 software [31]. The co-crystal native ligand was docked, and its resultant pose was uploaded to the PyMol software to superimpose it with the initial co-crystal native ligand pose. The command for aligning these two poses was given to PyMol 2.5.2 to calculate the RMSD. Lower RMSD values indicated better alignment and greater docking accuracy. Generally, RMSD values less than 2 Å represent valid docking protocols, whereas values greater than 4 Å indicate less accurate predictions [32,33].

#### 2.7.2. ADME-Toxicity.

The computational software SwissADME (http://www.swissadme.ch/index.php) was utilized for robust ADME predictions to assess *in vivo* biopharmaceutical parameters such as physicochemical properties, lipophilicity, water solubility, pharmacokinetics, and drug-likeness [20]. Additionally, PkCSM (https://biosig.lab.uq.edu.au/pkcsm/) and ProTox-3.0 software (https://comptox.charite.de/protox3/) were employed to precisely predict potential toxicity, including AMES toxicity, hepatotoxicity, nephrotoxicity, carcinogenicity, cytotoxicity, and mutagenicity [20]. The toxic doses of the compounds were expressed as the median lethal dose (LD_50_), which is the dose in mg/kg body weight at which 50% of the test subjects die upon exposure. The toxicity thresholds were categorized into various classes based on the LD_50_ values. Specifically, Class I is classified as fatal if swallowed (LD_50_ ≤ 5); Class II is considered fatal if swallowed (5 < LD_50_ ≤ 50); Class III is labeled as toxic if swallowed (50 < LD_50_ ≤ 300); Class IV is deemed harmful if swallowed (300 < LD_50_ ≤ 2000); Class V suggests that it may be harmful if swallowed (2000 < LD5_0_ ≤ 5000); and Class VI is classified as non-toxic (LD_50_ > 5000). This classification system helps assess the potential risks associated with exposure to different compounds (https://tox.charite.de/protox3/index.php?site=home) [34]....

### 2.8. Statistical analysis

Statistical analyses were performed using Microsoft Excel 2016 software. Each experiment was performed in triplicate, and the data are presented as the mean ± standard deviation. TPC, TFC, TCC, DPPH radical scavenging activity (IC_50_), and enzyme inhibitory activity (EC_50_) were determined by linear regression analysis.

## 3. Results and discussion

### 3.1. Extraction yield

A solvent system is essential for extracting the target bioactive compounds and their biological activities [35,36]. The *J. Marshi* rice is red in color and may contain anthocyanins, a colored flavonoid pigments that widely accumulate in colored-rice cultivars and exhibit several biological activities [9,11]. Likewise, several previous studies in the field of natural product chemistry have isolated bioactive phenolics and flavonoids from plants using 70% MeOH as a solvent because of their high solubility, penetration power, and partition capabilities [37–39]. Therefore, in this study, we aimed to extract polar bioactive compounds, including phenols, flavonoids, and anthocyanins, using 70% MeOH as the solvent. Heating at 55 °C during extraction improves efficiency by thermally breaking down the rice cell wall and increasing the solubility and release of intracellular constituents [20,35,40]. 70% methanol extract of *J. Marshi* rice produced a brown-red crude extract with a yield of 1.96%. Chanthathamrongsiri et al. [41] has observed extractive yield of 0.68% and 19.33% from grain and bran of black pigmented rice, respectively, using 75% ethanol for extraction. Similarly, extraction using 80% ethanol displayed % yield of 7.52 ± 1.13, 12.08 ± 1.81, and 4.88 ± 0.73 from black, red and brown rice landraces from north Thailand [42].

### 3.2. Phytochemical analysis

#### 3.2.1. Qualitative.

Phytochemical profiling indicated that phenols, tannins, flavonoids, anthocyanins, coumarins, saponins, steroids, terpenoids, alkaloids, carbohydrates, and proteins were present in the *J. Marshi* extract, whereas anthraquinone was absent (Table 1). Our findings are in line with several previous studies indicating the occurrence of phenolic acids, flavonoids, anthocyanins, and terpenoids in pigmented rice cultivars [9–11,43,44].

**Table 1:** Qualitative phytochemical analysis of *J. Marshi* rice extract from Nepal.

S.N.	Phytochemicals	Types of test	70% MeOH crude extract
1	Phenols	1. FeCl_3_ tests	+
2	Flavonoids	1. Alkaline reagent tests	+
		2. Shinoda tests	–
		3. Lead acetate tests	+
3	Tannins	1. FeCl_3_ tests	+
		2. Lead acetate tests	+
		3. Lime water tests	–
		4. Gelatin tests	–
4	Saponins	1. Foam tests	+
5	Alkaloid	1. Mayer`s reagent tests	+
		2. Wagner`s reagent tests	+
		3. Hager`s reagent tests	+
6	Terpenoids and steroids	1. Salkowski tests	+
7	Carbohydrates	1. Fehling`s tests	+
		2. Benedict`s tests	+
8	Protein/amino acids	1. Ninhydrin tests	+
9	Coumarin	1. NaOH tests	+
10	Anthocyanin	1. HCl/NH_4_OH test	+
11	Anthraquinones	1. Borntrager’s tests	–

(+) indicates presence; (–) indicates absence

#### 3.2.2. Quantitative.

The total phenolic, flavonoid, and carbohydrate contents were estimated using linear regression equations from the calibration curves of standard phenolic compounds: gallic acid (y = 0.0031 x + 0.02; R^2^ = 0.98), flavonoids: quercetin (y = 0.02 x −0.18; R^2^ = 0.97), and carbohydrates: D-glucose (y = 0.02 x + 0.31; R^2^ = 0.97). Results showed that *J. Marshi* has 68.44 ± 2.6 mg GAE/g, 122.32 ± 5.3 mg QE/g, and 874 ± 12.3 mg GE/g of dry extract. Stephen et al. found phenolic content ranging from 5.71 to 30.61 mg GAE/g and flavonoid content from 3.71 to 20.7 mg catechin equivalent/g in brown, red, and purple rice cultivars [11].

### 3.3. TLC profiling

**Fig 2:**
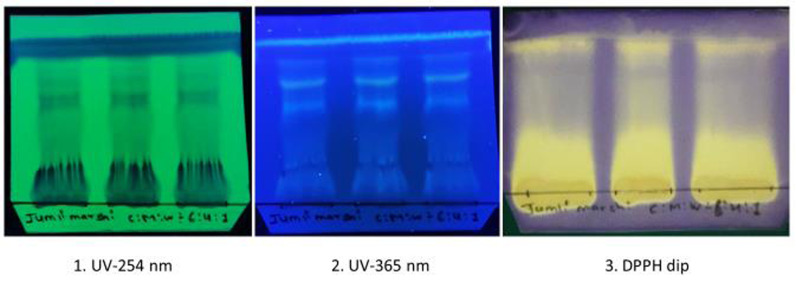
TLC profile of 70% MeOH extract from *J.* *Marshi* rice extract. Solvent system (CHCl_3_: MeOH: H_2_O = 6:4:1). Chromatogram visualized under 1.) short UV-254 nm; 2.) long UV-365 nm; and 3.) dipped in a 500 *µ *M DPPH solution.

Fig 2 illustrates the TLC profile of the 70% MeOH extract of *J. Marshi*. Several bands were observed on TLC when visualized under UV light, confirming the presence of UV-active phytochemicals such as phenols, flavonoids, and terpenoids. Furthermore, a yellow band on the violet/purple background appeared after dipping the developed TLC plate into the DPPH solution, suggesting the presence of antioxidants in *J. Marshi* rice [39].

### 3.4. LC-MS analysis

**Fig 3:**
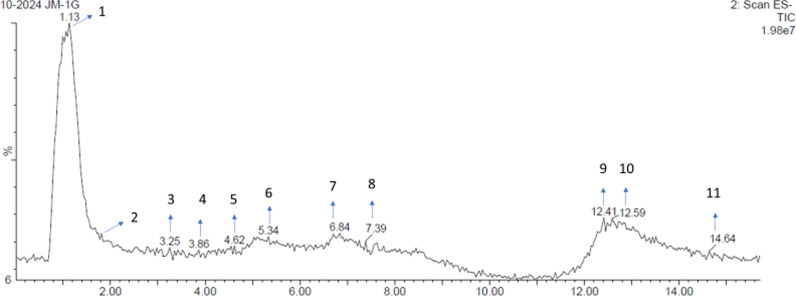
Liquid chromatography-mass spectra acquired in negative ion mode for 70% MeOH extract of *J.* *Marshi* rice from Nepal.

**Fig 4:**
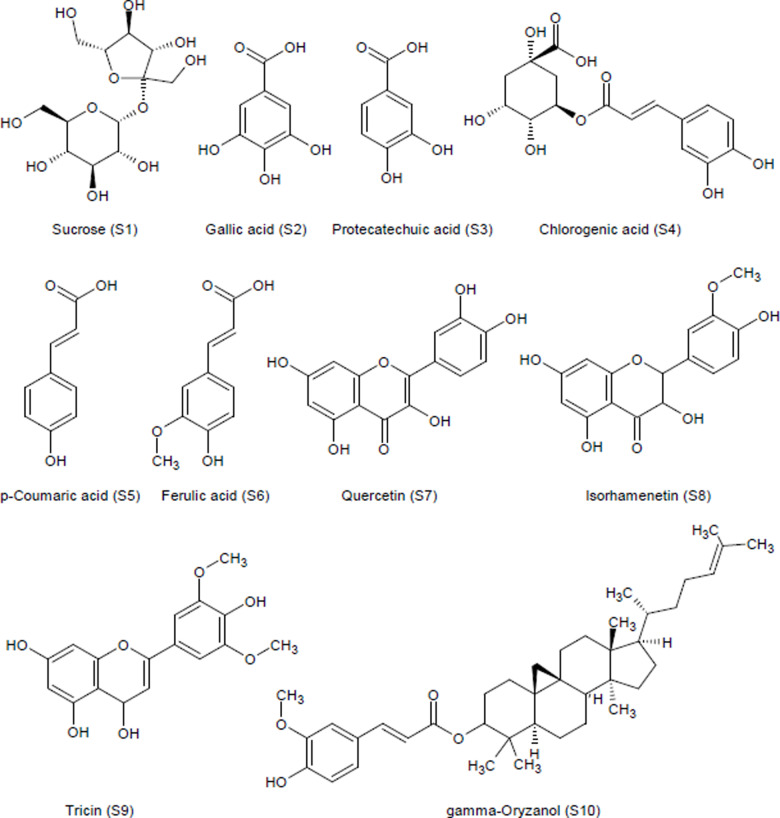
Chemical structures of phytoconstituents identified from the 70% MeOH extract of the *J*. Marshi rice cultivar.

Liquid chromatography-mass spectrometry (LC-MS) analysis of a 70% methanol (MeOH) extract of *J. Marshi* identified ten phytoconstituents, as detailed in Table 2. All compounds were characterized by analyzing their spectral information, including retention time and molecular mass, acquired using negative mode electron spray ionization-mass spectrometry (ESI-MS), as shown in Fig. 3 and 4.

**Table 2:** Phytoconstituents identified by liquid chromatography-mass spectrometry in 70% MeOH extract of *J. Marshi* rice.

Peak No.	RT (min)	Identification	Molecular formula	Actual *m/z*	Experimental *m/z* [M-H]	Adducts ion *m/z*	Fragments *m/z*
1	1.13	Sucrose	C_12_H_22_O_11_	342.3	341.4	377.39, 683.63	179
2	1.85	Gallic acid	C_7_H_6_O_5_	170.25	169.25	205.25, 339.35, 375.37	97.11, 113.15
3	3.25	Protocatechuic acid	C_7_H_6_O_4_	154.12	153.24	189.20, 307. 34	109.15
4	3.86	Chlorogenic acid	C_16_H_18_O_9_	354.31	353.42	389, 707.6	191
5	4.62	p-Coumaric acid	C_9_H_8_O_3_	164.16	163. 26	199.25, 327.42	–
6	5.34	Ferulic acid	C_10_H_10_O_4_	194.18	193.29	229.27, 387.5	–
7	6.84	Unknown	–	–	277.45	313.35, 555.5	165.16, 97.18
8	7.39	Quercetin	C_10_H_10_O_7_	302.23	301.32	337, 415, 603.23, 639, 905.42, 942	–
9	12.41	Isorhamnetin	C_16_H_12_O_7_	316.26	315.37	351.46, 419.25, 631.49, 667.50	285
10	12.59	Tricin	C_17_H_17_O_7_	330.23	329.57	365.66, 659.34	299.14, 271.29
11	14.64	gamma-Oryzanol	C_40_H_58_O_4_	602.9	601.1	637.99	–

RT, retention time in minutes. *m/z* represents the molar mass per unit charge.

Analysis of peak 1’s mass spectrum at 1.13 min retention time showed adduct ions with m/z values of 341.4 [M-H] ^−^, 377.39 [M + Cl] ^−^, and 683.63 [2M-H] ^−^. Additionally, a fragment ion at m/z 179 was observed, which resulted from the loss of hexose (162 Da) from the deprotonated ion, represented as [M-H-C_6_H_12_0_5_] ^−^. The compound was identified as sucrose by comparing these spectral data with previously published findings [18,45] and consulting mass spectra m/z databases, including Mass Bank of Europe (https://massbank.eu/MassBank/), IMPPAT (https://cb.imsc.res.in/imppat/), and National Library of Medicine (https://www.nlm.nih.gov/). This finding aligns with earlier reports on sucrose in rice cultivars [46].

The mass spectrum of the second peak eluted at 1.85 minutes, revealed adduct ions with m/z values of 169.25 [M-H]^ −^, 205.25 [M + Cl]^ − ^, 339.35 [2M-H]^ − ^, and 375.37 [2M + Cl]^ − ^. Additionally, a fragment ion was observed at m/z 97, which resulted from the sequential loss of CO_2_ (44 Da) and CO (28 Da) from the deprotonated ion, represented as [M-H-CO_2_-CO] ^−^. Through analysis of mass spectra m/z databases and relevant scientific literature [47], gallic acid was determined to be the compound corresponding to peak 2. This compound has been identified in several pigmented rice cultivars [9,10].

Analysis of the mass spectrum of peak 3 (retention time: 3.25 min) revealed adduct ions at m/z 153.24 [M-H]^ − ^, 189.20 [M + Cl]^ − ^, and 307.34 [2M-H]^ − ^. Furthermore, a fragment ion at m/z 109.15 was observed, resulting from the loss of CO_2_ (44 Da) from the deprotonated ion at m/z 153.24, was observed. By comparing these spectral data with the mass spectra m/z database and the relevant literature [48], peak 3 was determined to be protocatechuic acid. This compound has been previously identified in various rice grains [9,10].

The mass spectrum of peak 4 (retention time: 3.86 min) revealed adduct ions at m/z 353.4 [M-H]^ − ^, 389 [M + Cl]^ − ^, and 707.6 [2M-H]^ − .^ Furthermore, a fragment ion at m/z 191 was observed, which resulted from the loss of caffeoyl (C_9_H_6_O_3_; 162 Da) from the precursor ion at m/z 353.4. By comparison with the mass spectra m/z databases, including the Mass Bank of Europe, and examination of the relevant literature [18,49], peak 4 was determined to be chlorogenic acid. This compound has been previously identified in various rice landraces [9,10].

Analysis of peak 5’s mass spectrum, occurring at 4.62 min retention time, showed adduct ion with m/z values of 163.26 [M-H] ^−^, 199.25 [M + Cl] ^−^, and 327.42 [2M-H] ^−^. By comparing these spectral characteristics to previously identified phytoconstituents from rice varieties, we matched the compound with p-coumaric acid (m/z: 164.16). Based on this spectral similarity, peak 5 was tentatively identified as p-coumaric acid [9,10].

The analysis of the mass spectrum of peak 6 (elution time: 5.34 min) revealed adduct ions at m/z 193.29 [M-H]^ −^, 229.27 [M + Cl]^ − ^, and 387.5 [2M-H]^ − ^. A comparison of these spectral characteristics with previously identified phytochemicals from rice species suggested the presence of ferulic acid (m/z 164.16). Consequently, peak 5 was tentatively identified as ferulic acid based on findings reported in recent studies [9,10].

Mass spectrometric analysis of peak 7, which was eluted at 6.84 minutes, revealed adduct ions with m/z values of 277.45 [M-H] ^−^, 313.35 [M + Cl] ^−^, and 555.5 [2M-H] ^−^. Furthermore, fragmentation of precursor [M-H]^ −^ ions produced several fragment ions with m/z values of 175.21, 165.16, 159.11, 97.18, and 62.13. Despite these spectral data, no matching phytoconstituents were identified in any rice variety. Peak 7 was identified to be an unidentified compound.

The mass spectrum of peak 8 (elution time: 7.39 min) revealed several adduct ions: m/z 301.33 [M-H] ^−,^ 337 [M + Cl]^ − ^, 415 [M + CF_3_COO]^ − ^, 603.23 [2M-H]^ − ^, 639.4 [2M + Cl]^ − ^, 905.42 [2M-H]^ − ^, and 942 [3M + Cl]^ − ^. Furthermore, the low-intensity peak at m/z 285 indicates the removal of a hydroxyl group at the C-3 position in ring C, represented as [M-H-OH] ^−^. By consulting mass spectra m/z databases and relevant literature [50], peak 8 was identified as quercetin, a previously reported compound in pigmented rice cultivars [9].

Analysis of peak 9’s mass spectrum, occurring at 12.41 minutes retention time, exhibited a low intensity molecular ion peak at m/z 316.33 and various pseudo-molecular ions: m/z 315.37 [M-H] ^−^, 351.46 [M + Cl] ^−^, 419.25 [M + CF_3_COO] ^−^, 631.49 [2M-H] ^−^, and 667.50 [2M + Cl] ^−^. Additionally, a fragment ion at m/z 285.33 was observed, resulting from the loss of a methoxy group (OCH_3_; 31 Da) at C-4` from the deprotonated ion, is represented as [M-OCH_3_] ^−^. Compared with previously published spectral data [51] and the m/z mass spectra database, peak 9 was determined to be isorhamnetin, a compound previously identified in rice species [9].

The mass spectrum analysis of peak 10, which had a retention time of 12.59 min, revealed a molecular ion peak with low intensity at 330.54 and a quasi-molecular ion peak at m/z 329.57 [M-H] ^−^, 365.66 [M + Cl] ^−^ and 559.34 [2M-H] ^−^. Additionally, the [M-H]^ −^ ion generates daughter ions through the consecutive loss of two methyl free radicals (30 Da) and CO (28 Da) at m/z 299.14 [M-H-2CH_3_]^ −^ and 271.29 [M-H-2CH_3_-CO]^ − ^. Using information from mass spectra m/z databases, as well as relevant literature [52], peak 10 was identified as tricin, which was previously isolated from colored rice landraces [9].

The mass spectrum of peak 11 (retention time: 14.64 min) indicated adduct ions at m/z 601.1 [M-H] ^− ^ and 637.99 [M + Cl] ^− ^. By searching for the phytoconstituents that were previously isolated from rice varieties with similar spectral information, we found gamma oryzanol (m/z: 602.9) [8,9]; therefore, peak 11 was tentatively identified as gamma Oryzanol (Figs 3 and 4; Table 2).

### 3.5. *In vitro* bioassay

#### 3.5.1. Antioxidant activity.

**Fig 5:**
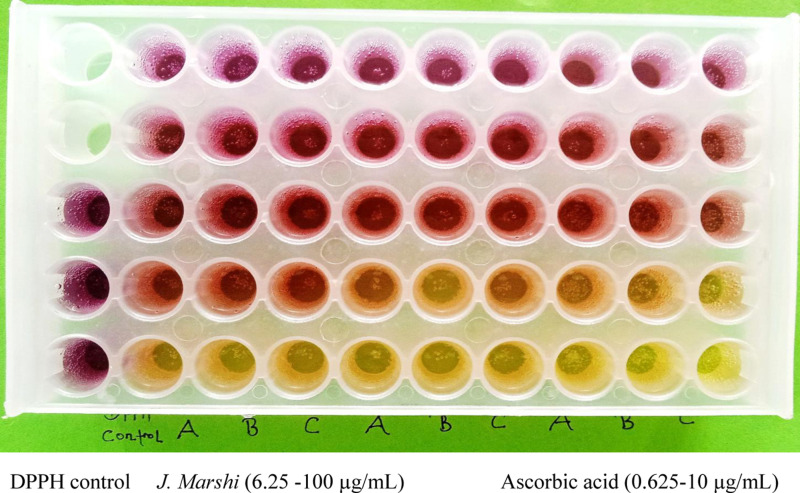
Evaluation of antioxidant activity of *Jumli Marshi* rice extract and standard ascorbic acid by DPPH free radical scavenging assay. The violet color represents the DPPH free radical (control), while the yellow color represents a higher concentration of *J. Marshi* extract or ascorbic acid scavenging the complete DPPH free radical.

**Fig 6:**
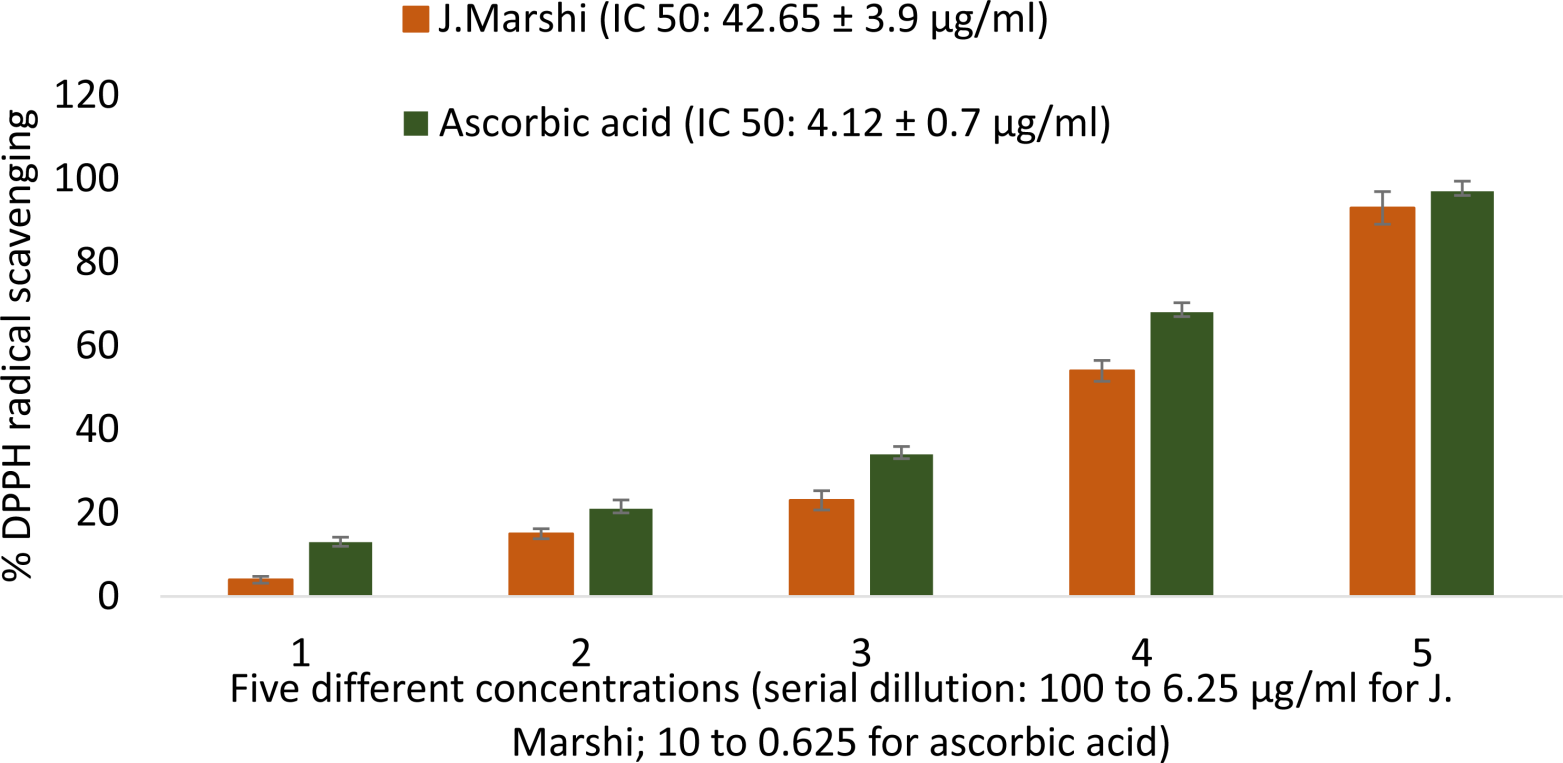
Dose dependent free radical scavenging activity of *J.* *Marshi* rice extract and standard ascorbic acid.

The antioxidant properties of *J. Marshi* rice extract was assessed using a DDPH free radical scavenging assay [53]. This method involves neutralizing the 2,2-diphenyl-1-picrylhydrazyl free radical by hydrogen atom donation, causing a color change from violet/purple to yellow, which was quantified by measuring absorbance at 517 nm (Fig 5). The extract neutralized DPPH free radicals proportionally to its concentration, with an IC_50_ value of 42.65 ±  3.9 µg/mL, while the standard ascorbic acid showed an IC_50_ value of 4.12 ±  0.7 µg/mL (Fig 6). In a previous DPPH assay, Katun et al. claimed that black rice has potent antioxidant activity with an IC_50_ value of 204.6 µg/mL, compared to white rice and brown rice, which showed IC_50_ values of 387.6 µg/mL and 859.38 µg/mL, respectively [54], indicating the superior antioxidant potential of current *J. Marshi* rice. Similarly, Ali et al. conducted research on pigmented rice cultivars, which reported greater DPPH scavenging properties for black rice (IC_50_: 25 µg/mL), followed by red rice (IC_50_: 32 µg/mL) and brown rice (IC_50_: 51 µg/mL). Glutenous black rice variety from Thai was found to scavenge DPPH free radicals with an IC_50_ value of 73.25 ±  0.85 µg/mL, compared to ascorbic acid, which yielded IC_50_ of 6.75 ±  0.46 µg/mL [41].

*J. Marshi* has strong antioxidant properties, as indicated by an IC_50_ value below 50 μg/mL [55], because of its diverse phenolic and flavonoid compounds such as tricin, quercetin, isorhamnetin, gamma oryzanols, ferulic acid, cinnamic acid, coumaric acid, protocatechuic acid, gallic acid, and chlorogenic acid [9]. These antioxidants in *J. Marshi* mitigates oxidative stress and protects the body against diabetes, premature aging, cancer, atherosclerosis, ulcers, gastrointestinal disorders, neurodegenerative diseases, coronary heart disease, hypertension, and liver toxicity [6,7]. Thus, *J. Marshi* rice is a beneficial source of functional foods.

#### 3.5.2. Antibacterial activity.

The global rise in antibiotic-resistant bacteria and emergence of new bacterial strains pose a significant threat to human health [56]. The misuse and overuse of antimicrobials have largely contributed to the increase in resistant bacteria, particularly *Escherichia coli*, *Klebsiella pneumoniae*, *Staphylococcus aureus*, *Pseudomonas aeruginosa*, *Acinetobacter baumannii*, and *Streptococcus pneumoniae* [57]. Additionally, the current rate of discovery and development of new antibiotics is insufficient to counteract growing resistance. Therefore, in addition to promoting antibiotic use, it is essential to prioritize the development of effective and safe alternatives.

**Fig 7:**
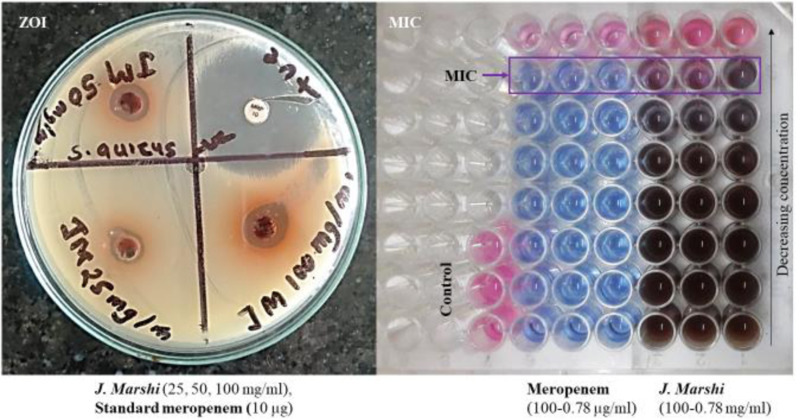
Photographs showing the antibacterial zone of inhibition (ZOI) and minimum inhibitory concentration (MIC) of *J.* *Marshi* and the standard meropenem against *S. aureus.* The clear diameter around each well of the Petri plate indicated the ZOI in millimeters. Likewise, the MIC indicated the lowest concentration of test extract or standard antibiotic showing no bacterial growth on visualization (blue color) against the negative control (pink), confirming bacterial growth.

The *J. Marshi* extract exhibited significant antimicrobial activity against *S. aureus* in the well diffusion assay, with inhibition zones of 7–11 mm compared to standard meropenem (24 ± 1.6 mm (Fig 7). The broth dilution method estimated a minimum inhibitory concentration (MIC) of 1.56 mg/mL and 1.56 µg/mL for *J. Marshi* and meropenem, respectively (Fig 7). However, *J. Marshi* did not exhibit antibacterial activity against *K. pneumoniae*, *P. aeruginosa*, and *E. coli* (Table 3). Recently, Burlacchini et al. observed the antibacterial activity of hydroxycinnamic and flavonoid-rich rice husk extracts against methicillin-resistant (MRSA) and methicillin-sensitive (MSSA) *S. aureus* clinical isolates, due to its inhibition of biofilm synthesis [58]. Likewise, in an antibacterial assay, Kundo et al. [59] reported effective antibacterial activity of rice bran extract against *Vibrio cholera* with a MIC value of 0.978 mg/mL, as well as weak antibacterial activity against *Salmonella spp*, *Shigella spp*, *Vibrio vulnificus*, and *Escherichia coli* with MIC values between 7.81 to 31.25 mg/mL.

**Table 3:** Antibacterial activity of *J. Marshi* rice extract using agar well diffusion and broth dilution protocols.

Bacterial strain	*J. Marshi*/standard antibiotics concentration	Antibacterial activity	
		ZOI (mm)	MIC (µg/mL)
*S. aureus*	100 mg/mL	11 ± 0.8	1560
	50 mg/mL	9 ± 1.2	
	25 mg/mL	7 ± 0.8	
	0 mg/mL	0	
	MP-10 µg	24 ± 1.6	1.56
*K. pneumonia*	100 mg/mL	0	–
	50 mg/mL	0	
	25 mg/mL	0	
	0 mg/mL	0	
	MP-10 µg	22.66 ± 1.2	1.56
*P. aeruginosa*	100 mg/mL	0	–
	50 mg/mL	0	
	25 mg/mL	0	
	0 mg/mL	0	
	MP-10 µg	17.33 ± 0.4	3.125
*E. coli*	100 mg/mL	0	–
	50 mg/mL	0	
	25 mg/mL	0	
	0 mg/mL	0	
	MP-10 µg	18 ± 0.8	3.125

MP-10 µg: Meropenem disc- 10 µg. ZOI: Zone of inhibition in mm, expressed as mean ±  SD. ‘-’ indicates not being tested.

The antibacterial properties of *J. Marshi* is likely due to its phenolic components, including tricin, quercetin, isorhamnetin, gamma-oryzanols, ferulic acid, coumaric acid, protocatechuic acid, gallic acid, chlorogenic acid, and their combinations [60–64]. Previous studies have shown that phenols and flavonoids exert antibacterial effects by disrupting cell membranes via hydrogen bonding [65]. These compounds also inhibit cell wall synthesis, enzyme production, and ATP formation, leading to bacterial death [60,63,66–68]. The lack of activity against *K. pneumoniae*, *P. aeruginosa*, and *E. coli* may be due to the impermeable lipopolysaccharide layer in the outer membrane of the gram-negative bacteria [69].

#### 3.5.3. Antidiabetic activity.

High-carbohydrate meals, particularly those rich in starch, are broken down by *α*-amylase and *α*-glucosidase into absorbable simple sugars, causing rapid blood sugar spikes that lead to diabetic complications [70]. Therefore, inhibition of these enzymes to reduce starch breakdown is a promising strategy for new diabetes treatments aimed at lowering postprandial blood glucose levels [30]. Previous scientific research has identified several compounds that inhibit *α*-amylase and *α*-glucosidase. For instance, acarbose, miglitol, and voglibose are used globally for the management of diabetes [28,71]. Accordingly, we also focused on these well-established targets for evaluating the antidiabetic potential of *J. Marshi* rice extract.

**Fig 8:**
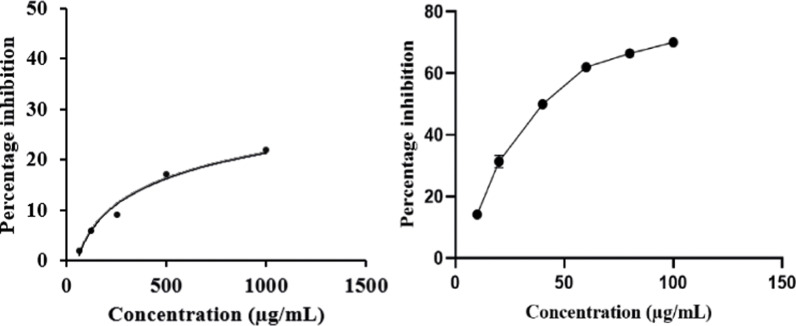
*α-*Amylase inhibitory activity of *J. Marshi* rice extract and standard acarbose.

**Fig 9:**
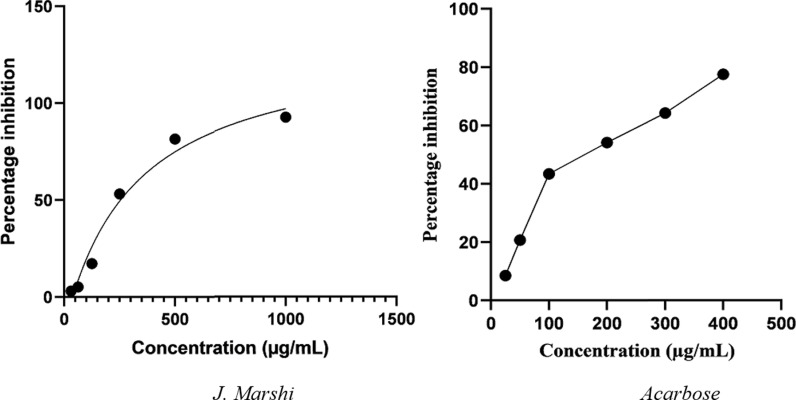
*α-*Glucosidase inhibitory activity of *J. Marshi* rice extract and standard acarbose.

In the current study, *α*-amylase hydrolyzed 2-chloro-4-nitrophenyl-α-D-maltotrioside (CNPG3) into 2-chloro-4-nitrophenol (CNP), 2-chloro-4-nitrophenyl-α-D-maltoside (CNPG2), maltotriose, and glucose [23,24]. The concentration of CNP (yellow color) was measured using a spectrophotometer at 405 nm wavelength. Significant enzyme inhibition led to reduced CNP production, resulting in lower absorbance. The *J. Marshi* extract inhibited *α*-amylase and *α*-glucosidase in a concentration-dependent manner. Its IC_50_ values ( > 1000 µg/mL and 250 ± 2.5 µg/mL) were compared to those of the standard acarbose (35.5 ± 1.5 µg/mL and 189.5 ± 1.9 µg/mL) as shown in Figs 8 and 9. A similar enzymatic study on pigmented rice cultivars found that brown, purple, and red rice bran exhibited no, weak, and moderate α-amylase inhibitory effects, respectively. In contrast, the study identified that purple and red rice bran demonstrated superior *α*-glucosidase inhibitory activity, with IC_50_ values of 8.44 μg/mL and 41.4 μg/mL, respectively, significantly much more potent than the standard acarbose (IC_50_: > 500 μg/mL) [11]. Shimoda et al. [16] demonstrated significant antidiabetic activity of purple rice extract by assessing its inhibitory potential on *α*-amylase and *α*-glucosidase, yielding IC_50_ values of 135 μg/mL and 409 μg/mL, respectively. Similarly, Chinese purple rice extract was found to suppress *α*-amylase and *α*-glucosidase activities, with IC_50_ values of 409 and 135 μg/mL, respectively [16]. The significant enzyme inhibitory activity of current *J. Marshi* rice extract must be due to the presence of phenolics, flavonoids, and terpenoids, including tricin, quercetin, isorhamnetin, gamma oryzanols, ferulic acid, cinnamic acid, coumaric acid, protocatechuic acid, gallic acid, chlorogenic acid, and their combinations, which have previously been reported in the literature for their antidiabetic properties [22,72].

### 3.6. *In silico* studies

#### 3.6.1. Molecular docking.

Molecular docking is a computational method used in drug discovery to improve the understanding of drug-protein interactions and facilitate the development of new therapies [26]. *α-*Amylase and *α-*glucosidase enzyme templates were chosen for the current *in silico* antidiabetic studies. The role of these endogenous enzymes in the pathogenesis and therapeutics of diabetes is well established [18,28,53] as they catalyze the breakdown of dietary starch into absorbable monosaccharides in the human gastrointestinal system, causing a rapid increase in blood glucose levels and subsequent diabetic complications [70]. Thus, the inhibition of *α*-amylase and *α*-glucosidase represents a promising strategy for the development of novel antidiabetic drugs [24,30]. The implementation of *in silico* approaches for the discovery and development of novel *α*-amylase and *α*-glucosidase inhibitors from a pool of natural products as antidiabetic drug candidates has gained significant traction in recent days [26,27,30].

In the current study, all the phytoconstituents (S1-S10) of *J. Marshi* were docked using the AutoDock Vina server to predict their binding site, binding energies, and orientation against *α*-amylase and *α*-glucosidase, which will support the effective *in vitro* inhibitory activity of these key antidiabetic enzymes as well as discover the molecular mechanisms underlying inhibition [22,25,26].

**Fig 10:**
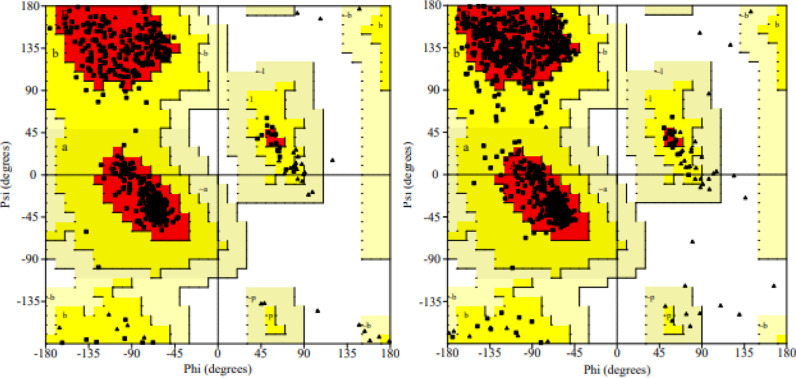
Ramachandran plot for the validation of target proteins. Amino acids (black dot), in red space (A, B, L) represented the most favorable residues, in the yellow zone (a, b, l, p) indicated the additional allowed residues, grey region (~a, ~ b, ~ l, ~ p) reflected the generously allowed residues, and white space indicated the disallowed residue stereochemistry for docking studies. Proline and glycine residues are depicted as triangles.

Validation of the target protein was performed through Ramachandran plot analysis (Fig 10), and The PROCHECK tool was utilized to generate the Ramachandran plot, which facilitates the assessment of the three-dimensional geometry of each amino acid residue and estimates the stereochemical quality of the protein [31]. The plot identified 495 amino acid residues in human pancreatic *α*-amylase and 850 residues in *α*-glucosidase. The analysis revealed 92.1% and 91.3% residues in the most favorable region, 7.9% and 8.7% in the additionally allowed region, and 0.0% and 0.0% in both the generously allowed and disallowed regions for *α*-amylase and *α*-glucosidase, respectively. The distribution of > 90% amino acids in the most favorable region, < 2% residues in generously allowed, and 0% residues in disallowed regions confirms the suitability and validity of the selected templates for modeling [73,74].

**Fig 11:**
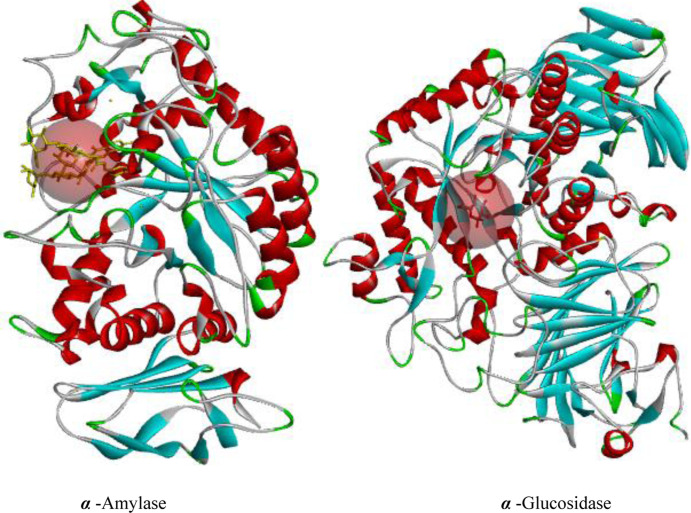
3D structure of the antidiabetic target proteins *α*-amylase and *α-*glucosidase. The co-crystal ligand bound at the catalytic pocket represented by a spherical grid.

Active site molecular docking was initiated using BIOVIA Discovery Studio, where the co-crystallized ligand was mapped to identify the catalytic triad and active residues within the 3D crystal structure of the host proteins (Fig 11). This visualization revealed the active residues ASP 197, GLU 233, and ASP 300 from α-amylase, and ASP 404, ASP 518, ARG 600, ASP 616, and HIS 674 from α-glucosidase, which are responsible for enzyme catalysis. These findings define the 3D space for site-specific molecular docking studies.

**Fig 12:**
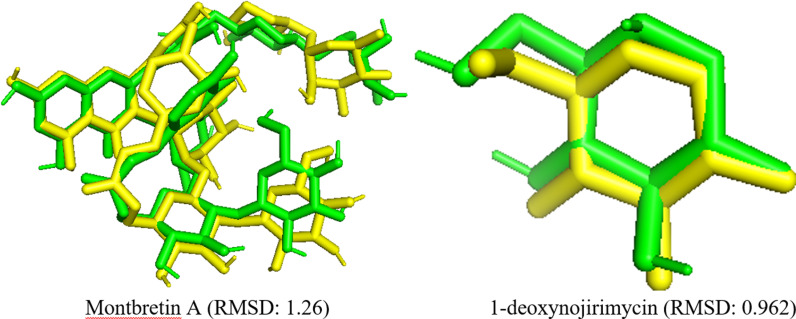
Validation of the docking protocol. Superposition of docked pose (green) and native pose (yellow) of co-crystal ligands, montbretin A and 1-deoxynojirimycin bound with *α*-amylase and *α*-glucosidase, respectively.

Molecular docking protocol validation is crucial for ensuring the accuracy, precision, and reliability of docking results [32]. RMSD values serve as the gold standard for assessing the validity of docking processes [32,75]. A lower RMSD value indicates greater docking accuracy. RMSD values less than 2 Å represent valid docking protocols, while values greater than 4 Å indicate less accurate predictions [32,33]. In the present docking analysis, an RMSD value of < 2 Å was achieved when the native and docked poses of the co-crystal ligand were superimposed in PyMol 2.5.2 (Fig 12), which verified the validity of the current docking protocols [32,75].

**Fig 13:**
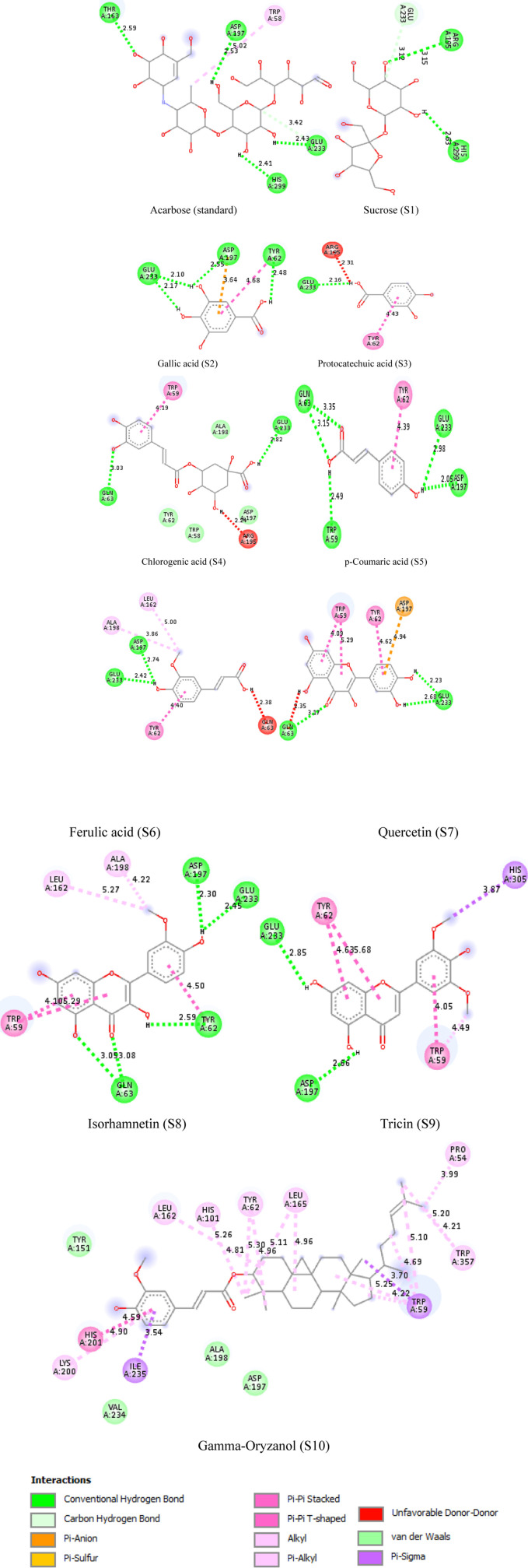
2D molecular interactions of J. Marshi phytoconstituents (S1-S10) and standard acarbose within the catalytic pocket of �-amylase.

**Fig 14:**
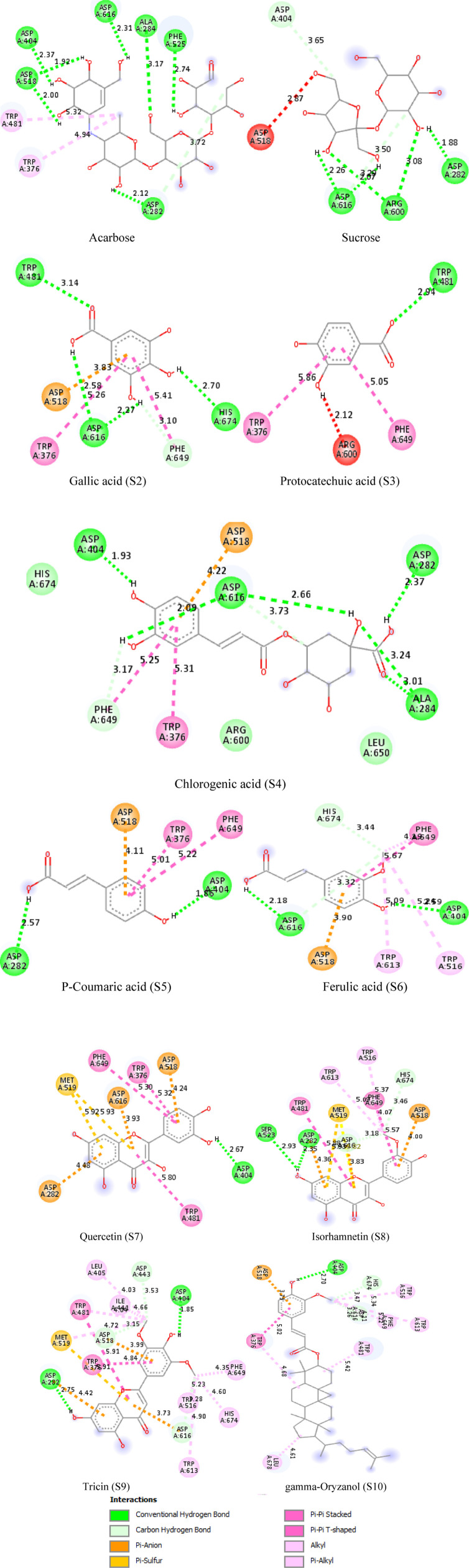
2D molecular interactions of J. Marshi phytoconstituents (S1-S10) and standard acarbose within the catalytic pocket of �-glucosidase.

**Fig 15:**
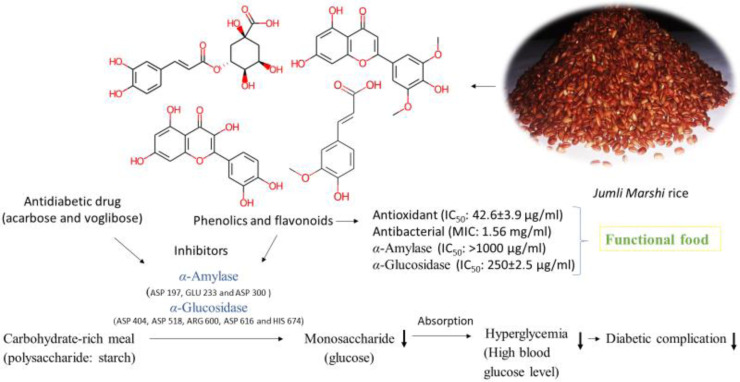
A systematic representation showing therapeutic potential of *J.* *Marshi* rice to validate their use as a functional food.

After docking, molecular interactions were prioritized based on their high negative binding energies, maximum hydrogen bonding, and minimal bond lengths. The phytoconstituents (S1 to S10) interacted with the enzyme’s catalytic residues, with binding energies ranging from −5.6 to −10.0 kcal/mol for *α*-amylase and −5.7 to −7.7 kcal/mol for *α*-glucosidase, compared to acarbose (−6.9 kcal/mol for *α*-amylase and −7.1 for *α*-glucosidase). The BIOVIA Discovery Studio Visualizer revealed that phytoconstituents formed bonds with catalytic residues through interactions such as hydrogen bonds, carbon hydrogen bonds, pi-sigma, pi-pi T-shaped, pi-donor hydrogen bonds, pi-alkyl, and π-π stacked interactions (Figs 13 and 14; Table 4). Conventional hydrogen bonding was predominant, indicating stable interactions [76]. Notably, gamma-oryzanol showed the most favorable docking energy of −10 kcal/mol for α-amylase, with multiple hydrophobic interactions. Chlorogenic acid (S4) had a significant binding affinity of −7.7 kcal/mol, forming five hydrogen bonds with α-glucosidase, close to primary catalytic residues ASP 404 and ASP 316. The strong enzyme binding of the rice phytoconstituents supports the *in vitro α*-amylase and *α*-glucosidase assays, explaining their inhibitory mechanism and therapeutic potential as scientific evidence to validate *J. Marshi* as a functional food product (Fig 15). Our studies suggest further molecular, cell line, and *in vivo* studies for comprehensive exploration of their therapeutic efficacy.

**Table 4:** Binding energy (kcal/mol) and molecular interaction of the *J. Marshi* phytoconstituents with antidiabetic targets *α* -amylase and *α* -glucosidase.

Ligands	*α* -amylase	*α* -glucosidase
	Docking score (active site residue interaction)	Docking score (active site residue interaction)
S1	−5.9 (GLU 233)	−6.1 (ASP 404, ASP 518, ARG 600, ASP 616)
S2	−5.6 (ASP 197, GLU 233)	−6.3 (ASP 518, ASP 616, HIS 674)
S3	−5.4 (GLU 233)	−6.1 (ARG 600)
S4	−7.8 (ASP 197, GLU 233)	−7.7 (ASP 404, ASP 518, ARG 600, ASP 616, HIS 674)
S5	−6.2 (ASP 197, GLU 233)	−5.7 (ASP 404, ASP 518)
S6	−6.5 (ASP 197, GLU 233)	−5.9 (ASP 404, ASP 518, ASP 616, HIS 674)
S7	−8.5 (ASP 197, GLU 233)	−7.2 (ASP 404, ASP 518, ASP 616)
S8	−8.7 (ASP 197, GLU 233)	−7.1 (ASP 518, ASP 616, HIS 674)
S9	−8.1 (ASP 197, GLU 233)	−7.2 (ASP 404, ASP 518, ASP 616, HIS 674)
S10	−10.0 (ASP 197)	−7.4 (ASP 404, ASP 518, ASP 616, HIS 674)
Acarbose	−6.9 (ASP 197, GLU 233)	−7.1 (ASP 404, ASP 518, ARG 600, ASP 616, HIS 674)

#### 3.6.2 . ADME−toxicity.

The prediction of absorption, distribution, metabolism, and excretion (ADME) parameters along with toxicity is crucial for the safety and efficacy of bioactive phytoconstituents in drug discovery and development pipelines [77]. The implementation of *in silico* approaches provides valuable and reliable forecasting of ADME-toxicity properties, offering significant advantages, including low cost, rapid analysis, eco-friendly predictions, and the ability to bypass animal testing [78].

Table 5 shows the ADME parameters for all identified phytoconstituents of *J. Marshi* rice species. Phytoconstituents, except S1, S4, and S7, showed TPSA values less than 140 Å^2^, indicating good absorption and better bioavailability [20]. Likewise, beside two phytoconstituents S1 and S7, all other obeyed the Lipinski’s rule of five (molecular weight: ≤ 500 g/mol; Log p_o/w_: ≤ 5; H-bond acceptor: ≤ 10; H-bond donor: ≤ 5; TPSA: ≤ 140 Å^2^) [34]; therefore, it satisfied the pharmacokinetic attributes, including solubility, membrane permeability and efficacy to be a good drug candidate [79].

**Table 5:** *In silico* ADME analysis of phytoconstituents (S1-S10) identified from *J. Marshi* rice from Nepal.

Physiochemical properties	S1	S2	S3	S4	S5	S6	S7	S8	S9	S10
Mol. Wt. (g/mol)	342.3	170.12	154.12	354.13	164.16	194.18	464.38	302.24	316.26	330.29
Number of rotatable bonds	5	1	1	5	2	3	4	1	2	3
Number of H-bond acceptors	11	5	4	9	3	4	12	7	7	7
Number of H-bond donors	8	4	3	6	2	2	8	5	4	3
Molar refractivity	68.16	39.47	37.45	83.5	45.13	51.63	110.16	78.03	82.5	86.97
TPSA (Å^2^)	189.53	97.99	77.76	164.75	57.53	66.76	210.51	131.36	120.36	109.36
**Lipophilicity**										
Log *P*_o/w_ (iLOGP)	0.85	0.21	0.66	0.87	0.95	1.62	0.94	1.63	2.35	2.58
Log *P*_o/w_ (XLOGP3)	−3.7	0.7	1.15	−0.42	1.46	1.51	0.36	1.54	1.87	3.07
Log *P*_o/w_ (WLOGP)	−5.4	0.5	0.8	−0.75	1.38	1.39	−0.54	1.99	2.29	2.59
Log *P*_o/w_ (MLOGP)	−4.37	−0.16	0.4	−1.05	1.28	1	−2.59	−0.56	−0.31	−0.07
Log *P*_o/w_ (SILICOS-IT)	−3.86	−0.2	0.26	−0.61	1.22	1.26	−0.59	1.54	2.06	2.59
Consensus Log *P*_o/w_	−3.29	0.21	0.65	−0.39	1.26	1.36	−0.48	1.23	1.65	2.15
**Water solubility**										
Log S (ESOL)	HS	VS	VS	VS	S	S	S	S	S	MS
Log S (Ali)	HS	S	S	S	S	S	MS	S	S	MS
Log S (SILICOS -IT)	S	S	S	S	S	S	S	S	S	MS
**Pharmacokinetics**										
GI absorption	Low	High	High	Low	High	High	Low	High	High	High
BBB permeability	No	No	No	No	Yes	Yes	No	No	No	No
p-gp substrate	Yes	No	No	No	No	No	No	No	No	No
CYP1A2 Inhibitor	No	No	No	No	No	No	No	Yes	Yes	Yes
CYP2C19 Inhibitor	No	No	No	No	No	No	No	No	No	No
CYP2C9 Inhibitor	No	No	No	No	No	Yes	No	No	No	Yes
CYP2D6 Inhibitor	No	No	No	No	No	No	No	Yes	Yes	Yes
CYP3A4 Inhibitor	No	Yes	Yes	No	No	No	No	Yes	Yes	Yes
Log *k*_*p*_ (Skin permeation; cm/s)	−11.02	−6.84	−6.42	−8.76	−6.26	−6.41	−8.88	−7.05	−6.9	−6.14
**Drug-likeness**										
Lipinski obey	No	Yes	Yes	Yes	Yes	Yes	No	Yes	Yes	Yes
Bioavailability Score	0.17	0.56	0.56	0.11	0.85	0.85	0.17	0.55	0.55	0.55
PAINS #alerts	0	1	1	1	0	0	1	1	0	0
Lead likeness	Yes	No	No	No	No	No	No	Yes	Yes	Yes
Synthetic Accessibility	5.16	3.65	1.07	4.16	1.61	1.93	5.32	3.23	3.26	3.12

HS: Highly soluble; VS: Very soluble; MS: Moderate soluble; S: Soluble.

In the context of *in silico* toxicity forecasting, all phytoconstituents demonstrated a safe profile in terms of Ames toxicity, hepatotoxicity, and cytotoxicity. In contrast, they exhibited a lower potential for inducing nephrotoxicity, with a maximum probability of 0.69, as shown in Table 6. In particular, chlorogenic acid (S4) and ferulic acid (S6) exhibited a high probability (P >  0.9) of causing immunotoxicity. Additionally, gamma-oryzanol displayed a high probability (P >  0.9) to behave as mutagens. Nevertheless, further molecular, cell line, genetic, and *in vivo* studies are required for the comprehensive validation of *in silico* safety claims.

**Table 6:** *In silico* toxicity prediction of phytoconstituents (S1-S10) of *J. Marshi* rice from Nepal.

Phytoconstituents	S1	S2	S3	S4	S5	S6	S7	S8	S9	S10
Ames toxicity	No	No	No	No	No	No	No	No	No	No
Oral rat acute toxicity (LD_50_) (mol/kg)	1.677	2.218	2.423	1.973	2.155	2.282	2.541	2.471	2.407	2.229
Max. tolerated dose human (log mg/kg/day)	1.574	0.7	0.814	−0.134	1.111	1.082	0.569	0.499	0.576	0.351
Predicted toxicity in rodent (LD_50_: mg/kg)	29700; C-6	2000; C- 4	2000; C- 4	5000; C- 5	2850; C- 5	1772; C- 4	5000; C- 5	159; C- 3	5000; C- 5	4000; C-5
Hepatotoxicity (probability in rodent)	− (0.96)	− (0.61)	− (0.59)	− (0.72)	− (0.51)	− (0.51)	− (0.82)	− (0.69)	− (0.72)	− (0.71)
Nephrotoxic (probability in rodent)	+ (0.67)	+ (0.69)	+ (0.61)	+ (0.56)	+ (0.66)	+ (0.62)	+ (0.76)	+ (0.62)	+ (0.64)	+ (0.64)
Cytotoxicity (probability in rodent)	− (0.70)	− (0.91)	− (0.90)	− (0.80)	− (0.81)	− (0.88)	− (0.69)	− (0.99)	− (0.95)	− (0.90)
Carcinogenicity (probability in rodent)	− (0.95)	+ (0.56)	+ (0.72)	− (0.68)	+ (0.50)	− (0.61)	− (0.85)	+ (0.68)	− (0.68)	− (0.69)
Immunotoxicity (probability in rodent)	− (0.98)	− (0.99)	− (0.99)	+ (0.99)	− (0.91)	+ (0.91)	+ (0.66)	− (0.87)	+ (0.58)	− (0.57)
Mutagenic (probability in rodent)	− (0.92)	− (0.94)	− (0.97)	− (0.93)	− (0.93)	− (0.96)	− (0.76)	+ (0.51)	− (0.94)	+ (0.91)

+ indicates Active; - indicates Inactive; C: Class of toxicity.

## 4. Conclusion

The 70% MeOH extract of *J. Marshi* rice was subjected to LC-MS analysis, revealing a variety of phenolic acids and flavonoids for the first time. Concurrently, *in vitro* bioassays have demonstrated that *J. Marshi* rice has antioxidant, antibacterial, and antidiabetic properties*. In silico* molecular docking studies further supported these findings by predicting notable interactions between *J. Marshi* phytoconstituents and catalytic residues of the target *α*-amylase and *α*-glucosidase enzymes. This scientific evidence validates the potential of *J. Marshi* rice as a functional food. The research concludes by proposing *J. Marshi* rice is a suitable alternative to nonpigmented rice for individuals with diabetes. However, further advanced research, including cell line and *in vivo* investigations of antioxidant, antidiabetic, antibacterial, and other bioactivities, are needed to validate and advance the current claims with great accuracy and precision.

## 5. Limitation of the study

We performed a tentative identification of phytoconstituents using LC-MS; therefore, we do not claim high precision or accuracy in our interpretations. This is first research study on the analysis of phytoconstituents and the investigation of *in vitro* and *in silico* biological activity of *J. Marshi* rice. Due to the lack of advanced laboratory setup and sophisticated technology, we were unable to isolate bioactive lead compounds and carry out cell line and *in vivo* studies, which may affect the current claims regarding the antidiabetic potential of *J. Marshi* rice species.

## Supporting information

S1 File Calibration curve, alpha amylase, alpha glucosidase, and mass spectra of *Jumli Marshi.* (DOCX)

## Abbreviations

*J. Marshi*: *Jumli Marshi*
LC-MS: Liquid Chromatography Mass Spectroscopy
GAE: Gallic Acid Equivalent
TLC: Thin Layer Chromatography
QE: Quercetin Equivalent
ADMET: Adsorption Distribution Metabolism Excretion And Toxicity
SD: Standard Deviation
TPC: Total Phenolic Content
TFC: Total Flavonoid Content.
