# Supplementary material for: LC-MS analysis and antioxidant, antibacterial, and antidiabetic activity of Jumli Marshi rice from Nepal: An in vitro and in silico investigation to validate their potential as a functional food
Source: PLoS One. 2025 Mar 10;20(3):e0319338. doi: 10.1371/journal.pone.0319338 (PMC11893129; doi:10.1371/journal.pone.0319338)
Supplement: S1 File — (DOCX) [file pone.0319338.s001.docx]

**LC-MS analysis and antioxidant, antibacterial, and antidiabetic activity of *Jumli Marshi* rice from Nepal: An *in vitro and in silico* investigation to validate their potential as a functional food**

Ram Kishor Yadav^1^_,_ Rekha Bhandari^1^, Harish Babu P C^2^, Prabhat Kumar Jha^3^, Bipindra Pandey^3*^, Sindhu KC^1^, Siddha Raj Upadhaya^4^, Sushil Panta^1^, and Sajan Lal Shyaula^5^, Khem Raj Joshi^1^

^1^School of Health and Allied Sciences, Pokhara University, Pokhara, Nepal

^2^Honeychem Pharma Analytical Services Private Limited, Bangalore, Karnataka, India

^3^Department of Pharmacy, Madan Bhandari Academy of Health Science, Hetauda, Nepal

^4^Central Department of Chemistry, Tribhuvan University, Kathmandu, Nepal

^5^ Nepal Academy of Science and Technology (NAST), Khumaltar, Lalitpur, Nepal

***** Corresponding author

Email: [bipindra.p101@gmail.com](mailto:bipindra.p101@gmail.com) (BP)

**Short Title:** LC-MS analysis and biological activity of *Jumli Marshi* rice

**Fig 1S. Calibration curve for standard gallic acid.**

**Fig 2S. Calibration curve for standard quercetin.**

**Fig 3S. Calibration curve for standard D-glucose.**


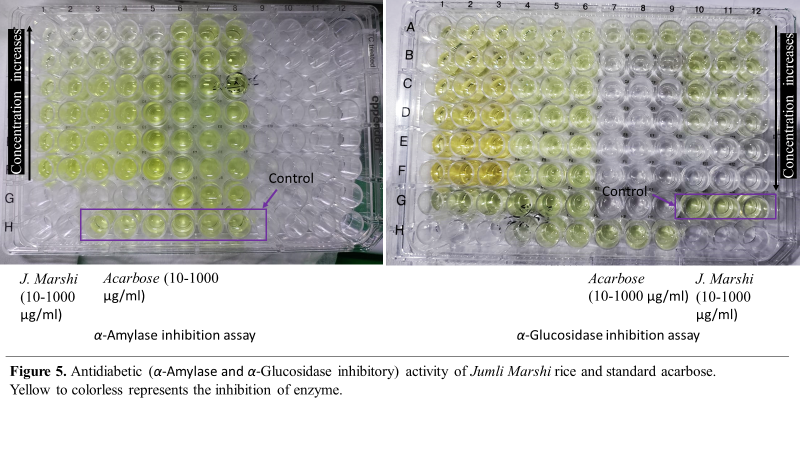


**Fig 4S. Antidiabetic (*α*-amylase and *α-*glucosidase inhibitory) activity of *J. Marshi* rice and standard acarbose.** Yellow to colorless represents the inhibition of enzyme.


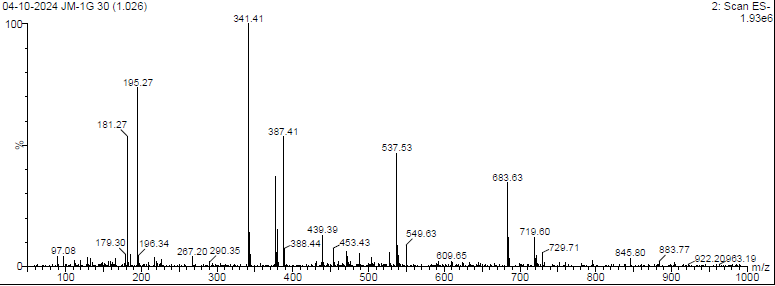


**Fig 6S. Mass spectra of sucrose acquired in negative mode ESI.**


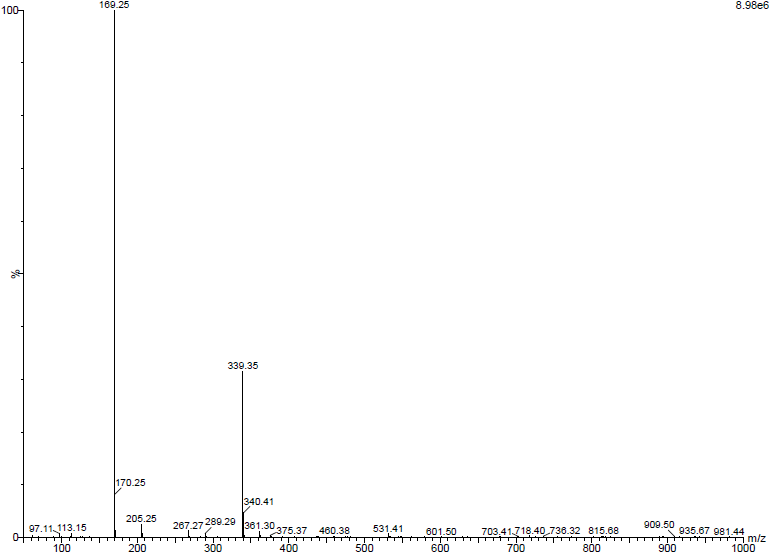


**Fig 7S. Mass spectra of gallic acid acquired in negative mode ESI.**


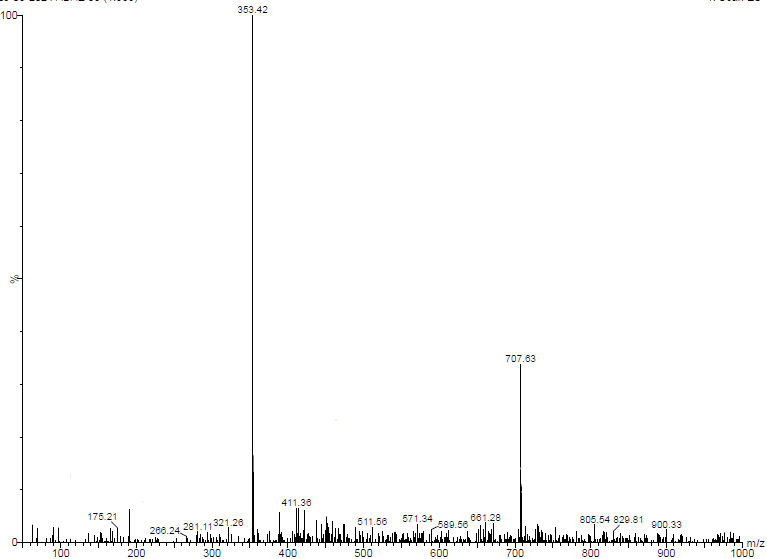


**Fig 8S. Mass spectra of chlorogenic acid acquired in negative mode ESI.**

**
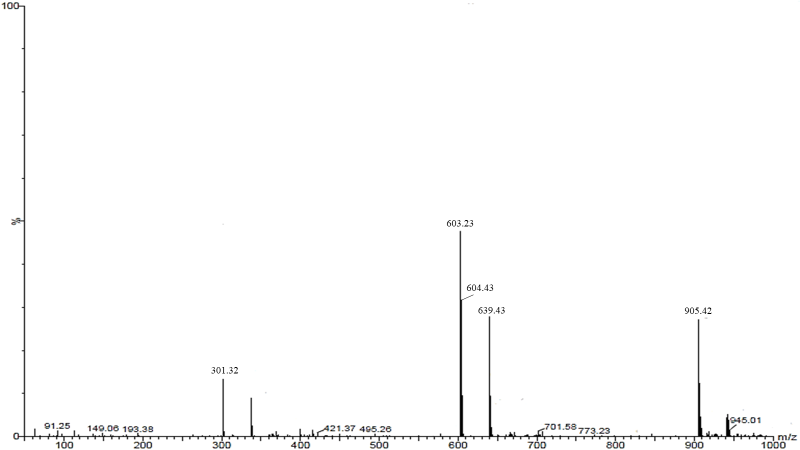
**

**Fig 9S. Mass spectra of quercetin acquired in negative mode ESI.**

**
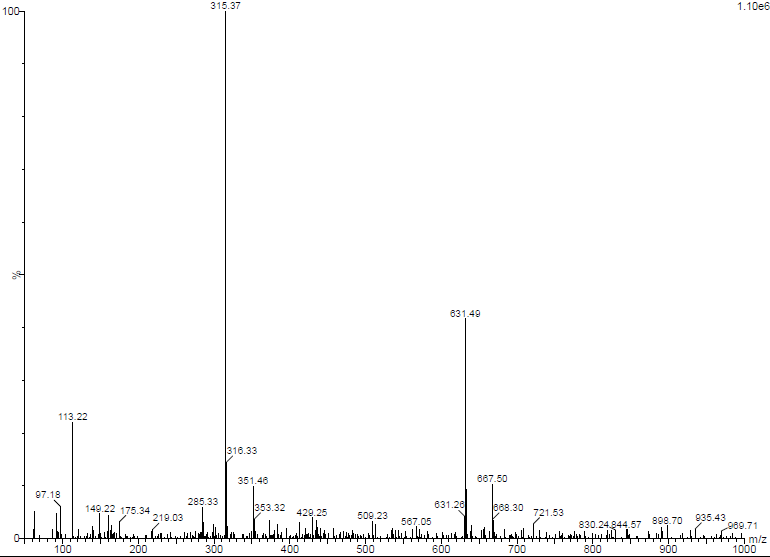
**

**Fig 10S. Mass spectra of isorhamnetin acquired in negative mode ESI.**
